# The Well-Being of Adolescents Conceived Through Medically Assisted Reproduction: A Population-Level and Within-Family Analysis

**DOI:** 10.1007/s10680-022-09623-6

**Published:** 2022-06-28

**Authors:** Hanna Remes, Maria Palma Carvajal, Riina Peltonen, Pekka Martikainen, Alice Goisis

**Affiliations:** 1 Population Research Unit, Faculty of Social Sciences, University of Helsinki, P.O. Box 18, 00014 Helsinki, Finland; 2 University College London, Social Research Institute, London, UK; 3 Department of Public Health Sciences, Stockholm University, Stockholm, Sweden; 4 Laboratory of Population Health, Max Planck Institute for Demographic Research, Rostock, Germany

**Keywords:** Medically assisted reproduction, IVF, Adolescence, Education, Home-leaving, Mental health

## Abstract

Medically assisted reproduction (MAR) plays an increasingly important role in the realization of fertility intentions in advanced societies, yet the evidence regarding MAR-conceived children’s longer-term well-being remains inconclusive. Using register data on all Finnish children born in 1995–2000, we compared a range of social and mental health outcomes among MAR- and naturally conceived adolescents in population-averaged estimates, and within families who have conceived both through MAR and naturally. In baseline models, MAR-conceived adolescents had better school performance and the likelihood of school dropout, not being in education or employment, and early home-leaving were lower than among naturally conceived adolescents. No major differences were found in mental health and high-risk health behaviours. Adjustment for family sociodemographic characteristics attenuated MAR adolescents’ advantage in social outcomes, while increasing the risk of mental disorders. The higher probability of mental disorders persisted when comparing MAR adolescents to their naturally conceived siblings. On average, MAR adolescents had similar or better outcomes than naturally conceived adolescents, largely due to their more advantaged family backgrounds, which underscores the importance of integrating a sociodemographic perspective in studies of MAR and its consequences.

## Introduction

Following the global trends of declining fertility rates, the postponement of childbearing, and the increased availability of infertility treatments, both the number and the proportion of children conceived through medically assisted reproduction (MAR) have been steadily increasing: to date, over eight million births have involved the use of assisted reproductive technology (Adamsson et al., 2019; Balbo et al., 2013; Schmidt et al., 2012). By 2015, the proportion of children conceived through assisted reproductive technology was between 3 and 5% in many European countries; and by 2017, this share had reached as high as 8% of all births in Denmark (De Geyter et al., 2020; Martins et al., 2018; Opdahl et al., 2020). Acknowledging the increasingly important role of MAR in the fertility of contemporary societies, an abundance of research has documented both similarities and differences between MAR- and naturally conceived children in various measures of early life outcomes (for a recent review, see Berntsen et al., 2019). Although modern reproductive technologies are considered very safe, birth outcomes are consistently poorer among children conceived through MAR. Whether the early health disadvantage originates from the MAR process itself or underlying factors such as parental subfertility remains unclear. Moreover, many important gaps in our knowledge about the link between MAR and children’s later outcomes remain. First, the literature on the longer-term well-being of MAR-conceived children is less well developed—most likely because of the relatively young ages of most MAR children to date and a lack of appropriate data—and the findings that exist are mixed and inconclusive (Bergh & Wennerholm, 2020; Berntsen et al., 2019; Hart & Norman, 2013; Rumbold et al., 2017; Wilson et al., 2011). Second, as most of these studies focus on specific domains of health and development, we lack a comprehensive understanding of the well-being of MAR children that is based on several health and social outcomes. Third, previous research has prioritized medical aspects around MAR, while less attention has been given to sociodemographic aspects, and the question of whether and how family background may influence differences between MAR and naturally conceived children.

Understanding how MAR- and naturally conceived children fare during late adolescence is important, as findings from the childhood period may not hold across the life course. While young people’s puberty-related physical development may already be slowing down in this phase, their neurocognitive and social and emotional development often continue well beyond adolescence (Dahl et al., 2018; Sawyer et al., 2012). Late adolescence is also a critical period for successfully transitioning to adulthood, with early transitions from school to employment and early home-leaving or parenthood often being associated with less advantageous long-term outcomes (Dorsett & Lucchino, 2014; Sirniö et al., 2017; Wickrama et al., 2010). The biological, cognitive, and behavioral changes that young people undergo while asserting their independence and developing their own identity also increase their vulnerability to mental health problems, self-harm, substance use, and other high-risk health behaviors that may have long-term effects on their later health and socioeconomic attainment (Akasaki et al., 2019; Dahl et al., 2018; Patton et al., 2016).

Using unique data from Finnish population registers, this study advances the literature on children conceived through MAR with three specific contributions. First, we compare MAR- and naturally conceived children on several social and mental health outcomes in late adolescence: school performance, secondary education track choice, school dropout rates, not being in education or employment (NEET), early home-leaving, antidepressant use, and health care episodes due to mental disorders or high-risk health behaviors. New to the existing literature, this comprehensive perspective allows us to develop a more nuanced understanding of how MAR-conceived children are doing and developing as they approach adulthood, and in what dimensions they are faring worse, the same, or better than naturally conceived children. Second, we examine the role of several observed child and parental sociodemographic characteristics, such as birth order, maternal age, and social background, as confounders that could affect the link between MAR and well-being. Third, for a subset of families who have conceived both through MAR and naturally, we compare the outcomes of MAR-conceived children to those of their naturally conceived siblings. This approach, which has not been previously used to study the longer-term outcomes of MAR children, enables us to control for all observed and unobserved factors shared between siblings, and to investigate whether there is an independent effect of being conceived through MAR on late adolescent outcomes.

## Background

### MAR and Offspring Health and Social Outcomes

The evidence that MAR-conceived children have poorer perinatal outcomes is well-established in the literature: Compared to naturally conceived children, children conceived through MAR are shown to be at increased risk of adverse birth outcomes, such as low birthweight, preterm delivery, and birth defects (Luke et al., 2021; Pinborg et al., 2013; Qin et al., 2017); and to be at higher risk of infant mortality (Rodriguez-Wallberg et al., 2020). However, despite these early life health disadvantages, the evidence regarding their physical development in childhood (Berntsen et al., 2019; Ludwig et al., 2006; Van Balen, 1998), as well as their cognitive (Barbuscia & Mills, 2017; Carson et al., 2011; Rumbold et al., 2017) and psychosocial development (Colpin, 2002; Van Balen, 1998; Wagenaar et al., 2008), is mostly reassuring. On average, MAR-conceived children have similar or better outcomes than naturally conceived children. By contrast, the evidence on the mental and behavioral health outcomes of MAR-conceived children is mixed and is less well developed. While most studies have found no or negligible associations between MAR and mental health or behavioral problems in childhood and adolescence (Hart & Norman, 2013; Wagenaar et al., 2009, 2011; Wilson et al., 2011; Zhu et al., 2011), some have detected increased risks of depression and binge drinking (Hart & Norman, 2013; Wagenaar et al., 2009). Moderately increased risks of having other specific psychiatric diagnoses, including autism spectrum disorder (Liu et al., 2017) and attention deficit/hyperactivity disorder (Kallen et al., 2011), have also been reported in previous studies (Berntsen et al., 2019; Rissanen et al., 2019). A Danish population-based study found small increases in the incidence of mental disorders among MAR children at ages 8–17 (Bay et al., 2013), while a longer follow-up study of children of women with fertility problems reported that these children had higher risks of experiencing mental disorders (Svahn et al., 2015). In a Finnish population-based study, MAR-conceived children followed up to young adulthood were shown to have a moderately higher risk of psychiatric disorders (Rissanen et al., 2019). In terms of cognitive outcomes such as IQ, learning abilities, and school performance, most studies have observed no major differences between MAR- and naturally conceived children, although a fairly recent review emphasized the need for caution, as most of these studies suffered from selection bias or failed to address confounding by family background (Rumbold et al., 2017). Recent high-quality population-based studies conducted in Denmark and Sweden found that MAR adolescents had better school performance, but after adjustment for confounders, the associations reversed, and small differences between MAR- and naturally conceived adolescents in favor of the latter emerged (Norrman et al., 2018, 2020; Spangmose et al., 2017). Given that the previous literature has focused on different dimensions of well-being (e.g., specific health or cognitive outcomes), a comprehensive assessment of how MAR-conceived children are doing as they grow older is still lacking.

One explanation for the mixed conclusions of the existing studies lies in their inclusion criteria: i.e., many of these studies used small or non-representative samples; and some excluded multiple births to increase the comparability of MAR children with naturally conceived children (Berntsen et al., 2019; Rumbold et al., 2017). Moreover, when studies are based on data from different time periods, comparing their findings can be difficult, since MAR techniques, as well as the prevalence and the diagnostics of the studied outcomes, have changed over time. Other plausible explanations for the conflicting results include the heterogeneity of the specific outcomes analyzed, the use of different methods, and differences in the adjustments for confounding and mediating factors. Many studies fail to show both unadjusted results showing how MAR children are actually doing compared to naturally conceived children, as well as adjusted results that aim to isolate the effect of the MAR treatment from those of confounding sociodemographic and health characteristics (Goisis et al., 2020). All of these issues, as well as the wide age range of children included in many studies (Ilioi & Golombok, 2015), make it difficult to compare and reconcile results across studies.

### Selection into MAR and Potential Mechanisms Behind the Differentials

The existing explanations as to how and why MAR might matter for offspring well-being can be divided into three broad categories. First, MAR procedures may result in physiological, metabolic, and endocrine changes that negatively influence the development of the offspring, and that can have long-term consequences (Barker, 2007; Bloise et al., 2014; Hart & Norman, 2013). The extent to which such influences relate to the MAR treatments per se, or to the underlying subfertility or other characteristics of the parents, remains unclear, but studies comparing MAR- and naturally conceived siblings lend support to the latter explanation (Berntsen et al., 2019; Goisis et al., 2019). Second, the often long process of conceiving through MAR and suffering from subfertility may be associated with higher levels of parental stress (Vahratian et al., 2011). Parents may become overprotective of their child and have unrealistic expectations, which could negatively influence the parent–child relationship and, as a consequence, the child’s psychosocial development (Bernstein, 1990; Colpin & Soenen, 2002; Golombok et al., 1995; Wagenaar et al., 2008, 2009). In contrast, other studies have shown that compared to families with naturally conceived children, MAR families have better parent–child relationships, higher levels of warmth, and more positive feelings related to parenting; possibly because MAR-conceived children are strongly desired (Goisis & Palma, 2021; Hahn & DiPietro, 2001; Wagenaar et al., 2008). Third, conception through MAR is not equally distributed in the population. Parents who give birth after MAR treatments tend to be older, are more often married and in stable relationships, have a higher level of education and are socioeconomically more advantaged than parents who conceive naturally (Chambers et al., 2014; Chandra et al., 2014; Goisis et al., 2020)—characteristics that are associated with favourable offspring outcomes in childhood as well as later in life (McLanahan, 2004). This selection into MAR is likely to play a significant role in differential outcomes between MAR and naturally conceived children, as higher parental education and income are known to predict higher cognitive development and educational attainment among their offspring (Feinstein, 2003; Heckman, 2006; McLanahan, 2004; Pfeffer, 2008). Moreover, having a more advantaged socioeconomic background is known to be associated with experiencing fewer mental disorders and psychosocial problems in childhood and adolescence (Lewis et al., 2014; Patalay & Fitzsimons, 2017; Reiss, 2013). In practice, behind any observed differences between MAR and naturally conceived children, there may be several potentially overlapping and interlinked mechanisms at play, with their relative importance and contribution varying across different outcomes.

### Study Hypotheses

Based on the previous literature, we expect to find that MAR-conceived adolescents face a trade-off, whereby they are less advantaged in terms of their health and psychosocial risk factors (following the potential negative influences of MAR procedures, parental subfertility, and the associated parental stress), but they are more advantaged than naturally conceived adolescents in terms of their parental social background. Thus, prior to adjustment for family characteristics, we expect similar or better social and educational outcomes among MAR than naturally conceived adolescents. In contrast, for the high-risk health behaviors and mental health outcomes, we expect a smaller advantage compared to the social outcomes or even disadvantage for MAR adolescents as the health and psychosocial risk factors relating to MAR are likely to have a more prominent role. Whether this will turn out to be the case is difficult to predict a priori, however, since family social background also matters for mental health outcomes (Reiss, 2013) and might offset any negative influences of MAR. Once we account for selection into MAR by controlling for sociodemographic factors, we expect the differences in social outcomes to attenuate and the differences in mental health outcomes to increase. For the within-family analyses that control for all observed and unobserved factors shared between siblings, we expect to find no major differences in the social outcomes, while any health or psychosocial risk factors relating to the treatment or parental stress might show in poorer mental health outcomes among siblings born after MAR. Using administrative data, we cannot fully separate between the different potential mechanisms at play beyond the assessment of the role of selection into MAR by sociodemographic factors. However, the within-family comparison enables controlling for additional unobserved family characteristics to the extent that they do not vary over time or between the siblings.

## Data and Methods

The study is based on longitudinal register data on a total population sample of Finnish women and all their biological children, for whom annual census data were linked with other administrative register data using personal identification numbers. In this study, we included children born in Finland between October 1, 1995, and December 31, 2000 (*n* = 303,922), who were part of the population in late adolescence, i.e., at ages 16–18 (*n* = 298,057). We focused on these birth cohorts because the medication data needed to identify MAR-conceived children were available from January 1, 1995, onward, and we could follow all of these children up to age 18 by the end of the follow-up period in 2018.

We excluded from the analyses births to mothers younger than age 20 (*n* = 5,371) or older than age 45 (*n* = 330) to increase our certainty that the women in our sample had undergone MAR treatment, instead of using fertility drugs for some other reason (e.g., menopause). Furthermore, we excluded 11,674 children (4.0%) due to missing data on any of the covariates (the highest prevalence of missing data for any individual covariate was 3.0% for maternal smoking). The final sample included 280,682 adolescents, except for the analyses on school performance and secondary education track choice, for which the analytical samples were smaller due to the availability of data on grade point averages (*n* = 219,624), and because some adolescents did not advance to secondary education (*n* = 269,662).

### Medically Assisted Reproduction (MAR)

The proportion of births attributable to MAR has been increasing steadily since the early 1990s in Finland, as well as in other Nordic countries (Opdahl et al., 2020). In Finland, both public health care and private clinics provide MAR services. While patients in the public sector pay a small fee for the services, with the rest of the costs being covered by the state-financed health care system for up to three cycles of treatment, patients in the private sector pay significantly more for their treatments, although the Social Insurance Institution reimburses about 60% of physician charges as well as purchases of prescription medication. During our study period (births 1995–2000), there were relatively small differences in access to MAR between socioeconomic groups with equitable use of public services, but women with higher socioeconomic position using private sector services more often (Klemetti et al., 2004). However, studies from Finland and Denmark have shown that compared to parents of naturally conceived children, parents who eventually conceive through MAR tend to be older, are more likely to be married, and have higher levels of education and income (Goisis et al., 2019; Wienecke et al., 2020). A similar pattern of selection into MAR with little change across time (1985–2014) was also reported in a recent study from Norway (Goisis et al., 2020).

We identified children conceived through MAR by linking information on women’s purchases of fertility treatment drugs with their child’s date of birth. The National Prescription Register maintained by the Social Insurance Institution covers prescription medication purchases from all retail pharmacies. The four main MAR techniques (ovulation induction, artificial insemination, IVF, and intracytoplasmic sperm injection (ICSI)) all involve use of fertility drugs. In order to identify women who were undergoing any of these treatments, we followed the method developed by Hemminki et al. (2003), which has been found to be reliable. We ignored cases of prescription medication purchases in the special reimbursement category, which indicates the use of fertility drugs to treat other diagnosed medical conditions, such as cancer.

In our analytical sample, 4.9% (*n* = 13,757) of adolescents had been conceived through MAR techniques including ovulation induction and artificial insemination, with 40% of them having been conceived through IVF. The analyses that involve comparing MAR adolescents to their naturally conceived siblings (born in the same family) include 4,923 adolescents (1.8% of the overall sample) born into 2379 families. The small number of siblings who did not have the same father (*n* = 45) are included in these analyses.

### Social and Mental Health Outcomes in Late Adolescence

With the aim of providing a comprehensive assessment of the longer-term well-being of MAR adolescents, we examine both social outcomes, including school performance and track choice, and their likelihood of dropping out of upper secondary education, of not being in education or employment, and of early home-leaving (outcomes 1–5), and their mental health outcomes, including antidepressant use, and health care episodes due to mental disorders or high-risk health behaviors (outcomes 6–12 below). All outcomes are measured in late adolescence (ages 16–18), a critical life period for successfully transitioning to adulthood, and for creating the conditions for longer-term outcomes.

In late adolescence, young people choose whether to continue their education, or whether to apply for a general (academic) or a vocational track that prepares them for specific professions, and that is often associated with short-term advantages and long-term disadvantages relative to pursuing an academic education (Brunello & Rocco, 2017). An individual’s school performance at the end of compulsory schooling strongly conditions both of these choices. Not completing any secondary education increases an individual’s likelihood of earning less than peers who graduate, and of being unemployed, on public assistance, and imprisoned (Christle et al., 2007). In particular, the experience of not being in employment, education, or training (NEET) has been shown to be associated with lower education and income (OECD, 2016), as well as with alcohol use disorders and poorer mental health (Manhica et al., 2019; O’Dea et al., 2014). Leaving the parental home is an important, and very concrete step toward independence in the transition to adulthood. Early home-leaving has been linked with various negative outcomes, such as lower educational attainment (White & Lacy, 1997) and youth poverty (Aassve et al., 2007; Ayllón, 2014), as well as health problems and excess mortality in early adulthood (Remes & Martikainen, 2012; Wickrama et al., 2010).

Half of all lifetime cases of mental disorders have been estimated to start by adolescence (Kessler et al., 2007), and mental disorders are the leading causes of health-related burdens among the young (Gore et al., 2011; Kieling et al., 2011). Adolescence is also a key period for the onset and establishment of many health behaviors (Hale & Viner, 2016), and high-risk health behaviors, such as substance use, tend to increase in late adolescence (Wiium et al., 2015). Having mental disorders or engaging in high-risk health behaviors during late adolescence may have long-lasting consequences on young people’s educational outcomes (Breslau et al., 2008), employment and social life (Wittchen & Hoyer, 2001), and later health (Akasaki et al., 2019; Patton et al., 2016).

#### Social Outcomes

1. Grade point average (GPA) measures school performance as the mean of theoretical subject grades (on a scale of 4–10) on the compulsory school completion certificate that is typically received at age 16. The grading is based on nationally determined learning goals, but does not involve standardized tests. Information on GPA was not available for the cohort born in 2000. Furthermore, information on GPA was missing for those individuals who never applied for upper secondary education (approximately 4% of each cohort), leaving us with data on GPA for 219,624 adolescents. We assessed GPA as a continuous variable.
2. Track choice (academic/vocational). In compulsory education, tracking is minimal, but upper secondary education divides into general (academically oriented) and vocational education. Among adolescents who had advanced to the upper secondary education level (96.1%), we predicted their likelihood of choosing the academic track at age 18, i.e., of completing a degree or being currently enrolled as a student on the academic track; with adolescents on the vocational track serving as the reference group.
3. Likelihood of dropping out of upper secondary education. Adolescents who had neither completed a secondary degree nor were enrolled as a student at the end of the year they turned age 18 were considered dropouts.
4. Likelihood of not being in education, employment, or training. NEET status was based on information on the main type of economic activity individuals were engaged in during the year they turned age 18, and included individuals who were unemployed, not in the labor market, and not students. Individuals in the military/civil service or on a disability pension were not included among the NEET.
5. Early home-leaving. Based on annually updated census information on household type, individuals who were not living with either of their parents at any time period between ages 16 and 18 were defined as early leavers.

#### Mental Health Outcomes

(6) Antidepressant use. Based on data from the National Prescription Register, we included any purchase of antidepressants at ages 16–18 with Anatomical Therapeutic Chemical (ATC) code N06A, including the combination product code N06CA01.
(7–11) Mental disorders were identified from admissions to inpatient or specialized outpatient care reported in the Finnish Health Care Register. Besides using an overall measure of any care episode due to mental disorders, we distinguished between internalizing (depression, anxiety), externalizing (conduct disorders, ADHD), and developmental disorders (including autism spectrum disorders). The different types of mental disorders (internalizing, externalizing, developmental, and other mental disorders) were identified using the International Classification of Diseases (ICD-10 codes in Appendix 4). We created five binary variables on whether the individual had at least one care episode due to any type of mental disorder, or any of the four specific types of mental disorders at ages 16–18.
(12) High-risk health behaviour was defined as any episode of inpatient or specialized outpatient care due to mental and behavioral disorders related to psychoactive substance use, poisoning by drugs, medications, or alcohol, intentional self-harm, or assault, based on the ICD-10 codes (Appendix 4).

### Confounders and Mediators

When comparing MAR and naturally conceived adolescents born in different families, we controlled for several observed child and parental characteristics that could confound the relationship between MAR conception and late adolescent outcomes: child sex (binary), birth year (categorical), birth order (categorical: 1, 2, and 3 or higher; the same birth order was assigned for twins/triplets), maternal age at birth (continuous and squared), and whether the mother smoked during pregnancy (binary). Furthermore, we included family structure at the time of birth (categorical: married, cohabiting, or single parents), the highest level of parental education in the household (categorical: basic, secondary, tertiary, and postgraduate education), and household income in deciles (categorical) from the year of birth. Finally, we included a measure of geographical area at birth based on the hospital districts in Finland (*n* = 20) to adjust for regional variation in MAR services (Klemetti et al., 2004).

As MAR children face a higher risk of adverse birth outcomes (Pinborg et al., 2013), which are negatively associated with later life outcomes (Boardman et al., 2002; Jefferis et al., 2002), we estimated additional models that included birth outcomes as potential mediators in the association between MAR and social and mental health outcomes in late adolescence. Using information obtained from the Finnish Medical Birth register, we included a binary indicator for low birth weight (LBW < 2500gr) and gestational age in days (continuous). As multiple births were more common in MAR pregnancies, we also included a binary indicator for multiple births (singleton vs. twins or triplets) among the potential mediators. Many previous studies have treated multiple births as a confounding factor, or restricted their analyses to singletons only. We did not exclude multiple births since they represent over a fifth of all MAR births in our sample, but carried out a sensitivity analysis on singletons only. Since our main focus was on the results adjusted for confounding by family background, the results from models including adjustment for potential mediators are shown in the appendix. While the existing research on gender-specific MAR associations is scarce (Punamäki et al., 2016), we tested for interactions by sex, as the prevalence and, potentially, the underlying mechanisms of outcomes such as depression and education are known to differ between males and females in late adolescence (Korhonen et al., 2017).

### Analysis

First, we describe the prevalence of the different social and mental health outcomes among MAR- and naturally conceived children in late adolescence. We used linear probability models to examine the differences by mode of conception both in the overall population, and in the sample of families with at least one MAR-conceived child and one naturally conceived child. We refer to the former analyses as the *between-family models,* as they compare children born in different families; and to the latter analyses as the *within-family models* (also known as sibling fixed-effects models), as they examine the association between MAR and well-being based on variation in the mode of conception between siblings born in the same family (Wooldridge, 2013). In order to account for potential confounders when comparing MAR- and naturally conceived children born in different families, the between-family models included controls for the observed child and parental characteristics. The within-family models fully account for the unobserved family-level confounding shared by siblings. Observed characteristics not shared by siblings, such as birth order, were adjusted for as in standard regression analyses. Our two approaches complement each other. While the between-family analyses allow us to obtain generalizable and externally valid results by analyzing the association between being conceived through MAR and adolescent outcomes in the overall population, the within-family analyses allow us to disentangle the effect of being conceived through MAR from all the observed and unobserved characteristics shared by siblings.

We estimated linear probability models on all of the binary outcomes in which the coefficients of the models are directly interpretable as marginal effects, and a linear model for GPA. For the binary outcomes, we used linear probability models (rather than logistic regression models) to avoid potential problems in comparing nested models and to include families where siblings do not vary on the dependent variable in the within-family estimations. The between-family models 1 and 2 show the association between MAR and each of the outcomes in the overall population, with model 1 adjusted only for the child’s sex and birth year and model 2 further adjusted for birth order, maternal age, maternal smoking, family structure, parental education, household income, and hospital district. The within-family models 3 and 4 present the fixed-effects estimations that compare siblings, with model 3 adjusted for the child’s sex and model 4 further adjusted for birth order, maternal smoking, and household income. We did not control for parental education and family structure, since they varied little between siblings.

We replicated the main analyses for IVF-conceived adolescents only (Appendix Table 5) to enable comparisons of our findings with those of many previous studies that have restricted their focus to IVF births. In order to assess the potential mediating role of multiple births, low birth weight, and lower gestational age among MAR-conceived children, we estimated the between- and within-family analyses while including both confounders and mediators (Appendix Table 6). We also replicated our main analyses for singletons only (Appendix Table 7). Finally, we tested for interactions by sex in our main analyses in order to examine possible heterogeneity in the associations between MAR and late adolescent outcomes (Appendix Tables 8 and 9).

## Results

### Descriptive Results

Table 1 shows the prevalence of all the outcomes by type of conception and analytical sample for the between-family and within-family analyses. For all of the social outcomes, MAR adolescents appeared to be more advantaged than their naturally conceived peers in the overall population. Compared to naturally conceived adolescents, MAR adolescents had better school performance (GPA 8.02 vs. 7.77); they were more likely to attend the academic than the vocational track in upper secondary education; they were less likely to drop out of school or be NEET (3.6 vs. 2.4%); and they were less likely to have left the parental home by age 18 (11 vs. 17%). The prevalence of antidepressant use (around 7.5%) and of care episodes due to any mental disorder (around 9%) in late adolescence were similar among MAR- and naturally conceived adolescents. Among the more specific mental health diagnoses, developmental disorders were slightly more common, but externalizing disorders were less common among MAR adolescents. Care episodes due to high-risk health behaviors were also less prevalent among MAR adolescents than naturally conceived adolescents (1.6 vs. 1.9%).

**Table 1:** Prevalence of social and mental health outcomes at ages 16–18 by type of conception in the analytical samples, children conceived naturally and through medically assisted reproduction (MAR) in Finland 1995–2000

	Between-family sample				Within-family sample			
	Naturally conceived		MAR		Naturally conceived		MAR	
	%	*n*	%	*n*	%	*n*	%	*n*
GPA (mean)	7.77^ab^	209,091	8.02^b^	10,533	7.94^a^	1,283	7.99	1,338
Academic track (ref. vocational)	51.85^ab^	256,320	62.52^b^	13,342	58.14^ac^	2,310	61.42^c^	2,372
School dropout	3.01^ab^	266,924	2.38^b^	13,758	2.30^a^	2,433	2.09	2,490
NEET status at age 18	3.64^ab^	266,924	2.42^b^	13,758	2.75^a^	2,433	1.97	2,490
Early home-leaving	17.14^ab^	266,924	11.08^b^	13,758	12.08^a^	2,433	10.56	2,490
Antidepressant use	7.58	266,924	7.46	13,758	7.15	2,433	6.91	2,490
Mental disorders (any)	9.28^a^	266,924	9.03	13,758	7.44^ac^	2,433	9.36^c^	2,490
Internalizing	5.92^a^	266,924	5.87	13,758	4.56^ac^	2,433	6.02^c^	2,490
Externalizing	1.28^b^	266,924	0.98^b^	13,758	0.99	2,433	1.16	2,490
Developmental	1.02^b^	266,924	1.24^b^	13,758	0.95	2,433	1.29	2,490
Other mental disorders	3.95	266,924	3.82	13,758	3.37	2,433	4.22	2,490
High-risk health behaviour	1.86^b^	266,924	1.56^b^	13,758	1.64	2,433	1.85	2,490

^a^Statistically significant difference between naturally conceived adolescents in between- and within-family samples at 95% confidence level. ^b^Statistically significant difference between MAR- and naturally conceived adolescents in the between-family sample at 95% confidence level. ^c^Statistically significant difference between MAR- and naturally conceived adolescents in the within-family sample at 95% confidence level. There were no significant differences between MAR adolescents in the between- and within-family samples

In contrast to the patterns in the between-family sample, the within-family comparison showed that MAR adolescents were more likely than their naturally conceived siblings to have mental disorders in late adolescence (9.4 vs. 7.4%). The gap between siblings was particularly pronounced in internalizing disorders. However, antidepressant use was equally prevalent among MAR adolescents and their naturally conceived siblings (6.9% vs. 7.2%). Care episodes due to externalizing, developmental, and other mental disorders, as well as high-risk health behaviors, were also found to be slightly more common among MAR adolescents; however, none of these differences were statistically significant. In terms of social outcomes, MAR adolescents were shown to have no consistent advantages compared to their naturally conceived siblings.

Overall, MAR adolescents had more advantaged family backgrounds than naturally conceived adolescents (Table 2). The parents of MAR-conceived adolescents were more likely to be married and had higher levels of education and household income. In addition, the mothers of MAR adolescents were, on average, older at the time of birth, and less likely to smoke during pregnancy. MAR adolescents were also more likely than their naturally conceived counterparts to be the first-born (89% vs. 77%). The parental characteristics of MAR adolescents were very similar in the between- and within-family samples, with maternal age being slightly lower in the within-family sample. However, MAR-conceived adolescents with naturally conceived siblings were somewhat less likely to be the first-born and the proportions of multiple births and adverse birth outcomes were lower than among MAR children in the between-family sample.

**Table 2:** Descriptive statistics for covariates by type of conception, children born in Finland 1995–2000

	Between-family sample		Within-family sample	
	Naturally conceived	MAR	Naturally conceived	MAR
Confounders				
Child's sex (male, %)	51.11	51.07	50.80	51.08
Maternal age at birth (mean)	29.98^ac^	32.38^bc^	31.74^ad^	30.85^bd^
* Birth order (%)*				
First-born	77.49^ac^	89.36^bc^	22.32^ad^	75.82^bd^
Second child	20.49^ac^	10.43^bc^	72.38^ad^	23.45^bd^
Third or higher	2.02^ac^	0.21^bc^	5.30^ad^	0.72^bd^
Maternal smoking (%)	14.83^ac^	7.06^c^	6.37^a^	7.39
* Family structure (%)*				
Married parents	63.72^ac^	77.53^bc^	80.76^a^	79.80^b^
Cohabiting parents	28.43^ac^	18.74^c^	16.03^a^	17.27
One-parent family	7.85^ac^	3.74^bc^	3.21^a^	2.93^b^
* Parental education (highest, %)*				
Higher tertiary	15.32^ac^	22.95^c^	22.73^a^	21.69
Lower tertiary	37.23^ac^	43.00^c^	43.03^a^	44.82
Secondary	40.22^ac^	30.54^c^	30.50^a^	30.20
Basic	7.23^ac^	3.50^c^	3.70 ^a^	3.29
Household income decile (mean)	5.52^ac^	6.90^bc^	6.27^ad^	6.74^bd^
Mediators				
* Type of Birth (%)*				
Singleton	97.8^ac^	77.55^bc^	98.44^ad^	88.67^bd^
Twins	2.17^ac^	21.39^bc^	1.44^ad^	10.81^bd^
Triplets	0.03^ac^	1.05^bc^	0.12^ad^	0.48^bd^
Low birth weight (%)	3.47^c^	14.15^bc^	2.88^d^	8.39^bd^
Gestational age (days, mean)	278.09^c^	271.10^bc^	277.62^d^	274.55^bd^

Figures in this table correspond to the mean of the analytical sample for the mental health outcomes (*n* = 280,682 in the overall population and 4,923 in the within-family sample). Covariates for birth year (1995–2000) and hospital district (*n* = 20) not shown

^a^ Statistically significant difference between naturally conceived adolescents in the between- and within-family samples at 95% confidence level. ^b^Statistically significant difference between MAR adolescents in the between- and within-family samples at 95% confidence level. ^c^Statistically significant difference between MAR- and naturally conceived adolescents in the between-family sample at 95% confidence level.^d^Statistically significant difference between MAR- and naturally conceived adolescents in the within-family sample at 95% confidence level

### Model Results

**Fig. 1:**
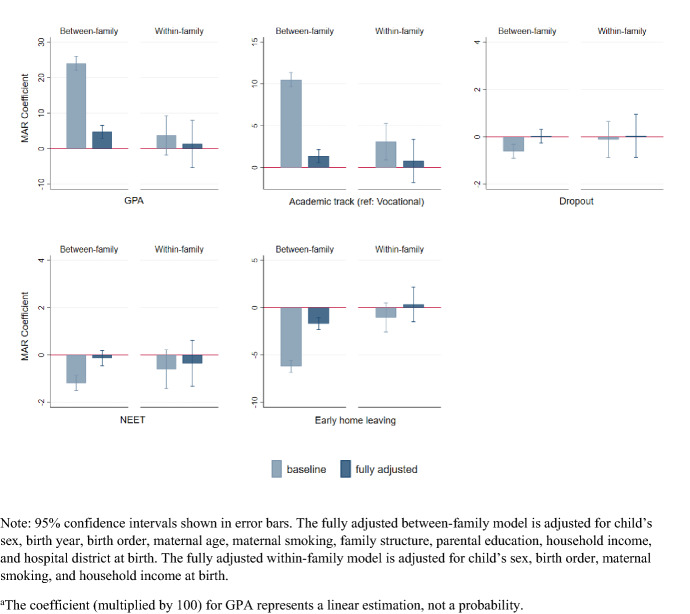
Differences in the probability (or the average level for GPA^a^) of social outcomes at ages 16–18 among children conceived by medically assisted reproduction (MAR) versus those conceived naturally. Between-family and within-family analyses of children born in Finland 1995–2000

**Fig. 2:**
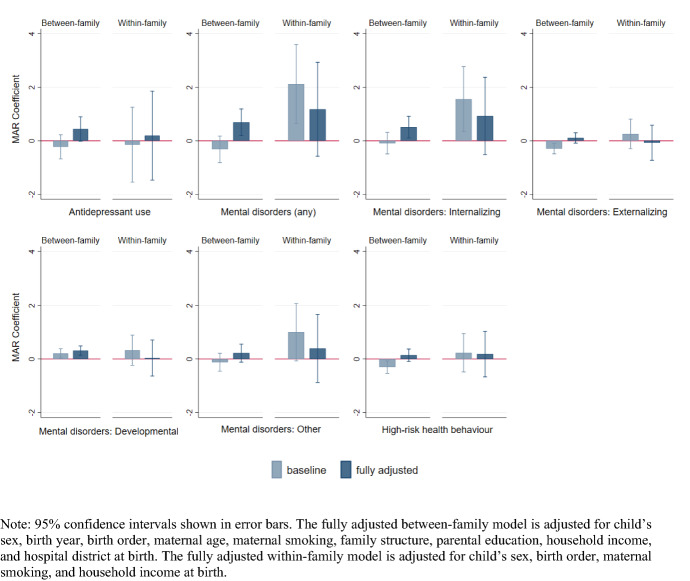
Differences in the probability of mental health outcomes at ages 16–18 among children conceived by medically assisted reproduction (MAR) versus those conceived naturally. Between-family and within-family analyses of children born in Finland 1995–2000

Table 3 shows the differences in the probability of experiencing all of the social outcomes (also shown in Fig. 1) and the mental health outcomes (Fig. 2) among MAR-conceived adolescents versus those conceived naturally in both the between-family and the within-family models.

**Table 3:** Differences in the probability (percentage points) of social and mental health outcomes at ages 16–18 among children conceived by medically assisted reproduction (MAR) versus those conceived naturally. Between-family and within-family analyses of children born in Finland 1995﻿–2000

MAR	Between-family sample			Within-family sample		
	Model 1	Model 2	*n*	Model 3	Model 4	*n*
Grade point average (GPA)^a^	24.04*** (1.008)	4.763*** (0.925)	219,624	3.732 (2.809)	1.295 (3.397)	2621
Academic track (ref. vocational)	10.50*** (0.436)	1.369*** (0.405)	269,662	3.081** (1.107)	0.786 (1.324)	4682
School dropout	− 0.611*** (0.149)	0.026 (0.150)	280,682	− 0.114 (0.388)	0.040 (0.462)	4923
NEET status at age 18	− 1.184*** (0.163)	− 0.137 (0.163)	280,682	− 0.603 (0.417)	− 0.357 (0.496)	4923
Early home-leaving	− 6.201*** (0.325)	− 1.695*** (0.318)	280,682	− 1.040 (0.786)	0.342 (0.935)	4923
Antidepressant use	− 0.227 (0.230)	0.440 (0.232)	280,682	− 0.146 (0.713)	0.193 (0.848)	4923
Mental disorders (any)	− 0.319 (0.252)	0.693** (0.254)	280,682	2.124** (0.752)	1.177 (0.895)	4923
Internalizing	− 0.090 (0.205)	0.511* (0.207)	280,682	1.556* (0.620)	0.928 (0.738)	4,23
Externalizing	− 0.297** (0.098)	0.106 (0.099)	280,682	0.257 (0.282)	− 0.072 (0.335)	4923
Developmental	0.209* (0.088)	0.315*** (0.090)	280,682	0.329 (0.288)	0.037 (0.343)	4923
Other mental disorders	− 0.120 (0.170)	0.220 (0.172)	280,682	1.002 (0.546)	0.395 (0.649)	4923
High-risk health behaviour	− 0.304** (0.118)	0.140 (0.119)	280,682	0.234 (0.365)	0.185 (0.435)	4923
Adjustments:						
Child's sex	x	x		x	x	
Birth year	x	x				
Birth order		x			x	
Maternal age at birth		x				
Maternal smoking		x			x	
Parental education at birth		x				
Family structure at birth		x				
Income decile at birth		x			x	
Hospital district at birth		x				

Standard Errors in parentheses. Sibling fixed effects used for the within-family models. Significance levels: ***p < 0.001, **p < 0.01, *p < 0.05. ^a^The coefficient (multiplied by 100) for GPA represents a linear estimation, not a probability

In the between-family baseline models, all of the social outcomes were more favorable for MAR adolescents. This consistent advantage over naturally conceived children was mostly or fully attenuated when we adjusted for sociodemographic characteristics. In the adjusted models (model 2), the likelihood of dropping out of school or being NEET no longer differed, but the MAR adolescents still had a marginally higher GPA, and they remained slightly more likely to attend the academic track in upper secondary education (1.4 percentage points), and less likely to have left the parental home early (1.7 percentage points). In the mental health outcomes, the baseline models (model 1) largely showed no differences or a modest advantage for MAR-conceived adolescents. However, when adjusted for child and parental characteristics (model 2), the lower probability (0.3 percentage points) among MAR adolescents of having both externalizing disorders and high-risk health behaviors disappeared. Moreover, after adjustment for the sociodemographic characteristics, MAR-conceived adolescents were found to be more likely to have received care for any mental disorder (0.7 percentage points), and for internalizing disorders in particular. As an exception to the pattern of reversed associations, the probability of having care episodes due to developmental disorders was moderately higher among the MAR adolescents than among the naturally conceived adolescents in both the baseline and the adjusted models.

The baseline models in the within-family analyses (model 3) showed a markedly higher probability of having any mental disorder (2.1 percentage points) among MAR adolescents. This discrepancy between MAR- and naturally conceived siblings was largely due to internalizing disorders, although other mental disorders also contributed to the overall difference. In social outcomes, MAR adolescents were more likely to attend the academic rather than the vocational track in upper secondary education. Otherwise, however, there were no consistent differences by mode of conception between siblings. After adjustment for birth order, maternal smoking, and income decile at birth (model 4), all of the differences between the MAR- and naturally conceived siblings were fully attenuated or were no longer statistically significant. Among these factors, birth order appeared to be the most important. However, given that over 75% of the MAR-conceived children in our data were first-born (Table 2), separating the effects of MAR and birth order is difficult.

### Sensitivity Analyses

We adjusted the models for low birth weight, gestational age, and multiple birth, which might mediate the association between being conceived through MAR and later health and social outcomes. However, the results (models 2a and 4a, Appendix Table 6) did not differ greatly from those of the main adjusted models (models 2 and 4, Table 3). The higher probability of antidepressant use, of having internalizing and developmental mental disorders, and of having a higher GPA and choosing the academic track, as well as the lower probability of leaving home early, observed among MAR adolescents in the main models all remained in the between-family models further adjusted for birth outcomes. By contrast, no statistically significant differences were observed between the MAR adolescents and their naturally conceived siblings after adjustment for birth outcomes. We also replicated the models excluding multiple births, and the results among singletons remained essentially the same (Appendix Table 7).

Similar to the full sample of MAR adolescents, the subsample of IVF-conceived children had better social outcomes, though this difference was attenuated when we adjusted the models for sociodemographic factors (Appendix Table 5). Except for higher antidepressant use, the mental health outcomes among IVF adolescents were similar to those of the naturally conceived adolescents in the baseline models. However, following adjustment for sociodemographic factors, only developmental disorders were shown to be more common among IVF- than naturally conceived adolescents, but the pattern of attenuating or reversed associations was nevertheless similar between the two groups. In the within-family analyses, the sample lost much of its statistical power. Further adjustment for LBW, gestational age, and multiple births (Appendix Table 6) had no major effects on the estimates in the between-family or the within-family models.

We tested for interactions to examine possible heterogeneity in the associations between MAR and late adolescent outcomes by sex (estimates based on the interaction models shown separately for boys and girls in Appendix Tables 8 and 9). In the between-family models, there were no interactions of major importance between MAR and sex. Compared to girls, MAR-conceived boys were slightly less likely to have ‘other’ mental disorders than boys in general, to be NEET, or to drop out of secondary education, but they were more likely to leave home early. In the within-family analyses, MAR-conceived girls were shown to be more likely to drop out of school, but their probability of having developmental disorders was clearly lower. The results of the within-family analyses by sex need to be interpreted cautiously, however, due to the small number of cases.

## Discussion

Given the increasingly important role that MAR is playing in the realization of fertility intentions in contemporary societies, many studies have analyzed the potential differences and similarities between MAR- and naturally conceived children. While most of the prior studies on MAR-conceived children have focused on birth outcomes and childhood, a small number of population-based studies have been able to extend the follow-up period into adolescence and young adulthood (Berntsen et al., 2019; Norrman et al., 2018; Rissanen et al., 2019; Svahn et al., 2015). This study provides the first comprehensive assessment of MAR children’s well-being during the critical life period of late adolescence when experiencing health problems and social disadvantage can reduce young people’s chances of successfully transitioning to adulthood, with long-term consequences for their later health and well-being. We analyzed and compared several outcomes among MAR- and naturally conceived adolescents in both population-averaged estimates, and within families who had at least one child conceived through MAR and one child conceived naturally, paying particular attention to selection on sociodemographic characteristics.

### Comparison to Previous Studies

In line with our hypotheses, we found that MAR adolescents fared better than their peers in all of the social outcomes analyzed, a finding largely explained by their more advantaged family backgrounds. Moreover, the within-family analyses, which controlled for all factors shared between siblings, found no consistent advantages among MAR adolescents. Our findings on educational outcomes corroborate the few recent high-quality studies that uncovered no meaningful differences in school performance or in the transition to secondary education between MAR- and naturally conceived adolescents after adjustment for family sociodemographic characteristics (Norrman et al., 2018, 2020; Spangmose et al., 2017). Apart from research on education, there is little prior research on social outcomes among MAR-conceived adolescents. Adding to the existing evidence, our results detected a qualitative difference in educational choices: i.e., that MAR adolescents were more likely to attend an academic rather than a vocational track in secondary education, which is associated with a higher probability of advancing to tertiary education, and of having better employment opportunities over the long term (Hampf & Woessmann, 2017). Among our other novel findings were that MAR adolescents had a lower probability of not being in education or employment and of leaving home early. The lower likelihood of early home-leaving observed among MAR adolescents might reflect parental protectiveness, the overall closeness of the parent–child relationships (Goisis & Palma, 2021; Ilioi & Golombok, 2015), and, possibly, having a more stable home environment with parents who are older and more likely to be married than average. Overall, our findings underscore the crucial role of sociodemographic selection in explaining the advantages of MAR adolescents in social outcomes observed at the population level. MAR-conceived children on average grow up in resourceful environments, which is strongly associated with better educational and social outcomes (Bloome, 2017; Pfeffer, 2008; Triventi, 2013).

In terms of mental health outcomes, the MAR adolescents had no clear advantages from the outset, contrasting our findings on the social outcomes—consistent with our expectation. Moreover, adjustment for observed family sociodemographic characteristics showed that they were at higher risk of having mental health problems, internalizing disorders in particular, confirming the hypothesis on their more advantaged social background compensating for the otherwise increased risks. Our findings are broadly in line with the evidence emerging from previous population-based studies from Denmark (Bay et al., 2013; Svahn et al., 2015) and Finland (Rissanen et al., 2019), despite our focus on late adolescence, rather than on lifetime prevalence during childhood and adolescence. Our results take these existing findings further by testing and showing the persistence of the effects in the within-family models, and thus highlight the potentially independent effect that MAR has on adolescents’ mental health.

While overall measures of mental health problems are likely to mask important differences, previous studies on specific mental health outcomes have provided somewhat inconsistent results, perhaps due to selected study samples, relative rareness of the conditions, differing methodological approaches, and differences in the etiology and onset of specific mental disorders. Our findings indicating that MAR adolescents have a higher risk of internalizing disorders and slightly increased antidepressant use have no direct point of reference, but a few prior studies have reported that MAR adolescents experience higher levels of anxiety (Rissanen et al., 2019), depression (Hart & Norman, 2013; Wagenaar et al., 2009), and affective disorders (Svahn et al., 2015). The moderate, but consistently higher, risk of having developmental disorders we found among MAR adolescents is in line with a recent review concluding that MAR conception is associated with autism spectrum disorders (Liu et al., 2017). In contrast to previous results showing a slightly increased risk of ADHD and conduct disorders among MAR children (Bay et al., 2013; Kallen et al., 2011; Svahn et al., 2015), we found no differences in externalizing disorders, but this discrepancy could be partly explained by our focus on late adolescence, which might ignore cases with early onset. As a new addition to the existing literature, we found no differences in the health consequences of high-risk health behaviors that often emerge in mid- and late adolescence (Akasaki et al., 2019; Hale & Viner, 2016).

Different mechanisms could underlie the higher probability of mental disorders we observed among MAR adolescents. First, having difficulties conceiving and undergoing the often long and stressful MAR process may have exposed the parents to mental health problems, such as depression and anxiety, which may, in turn, have put the children themselves at higher risk of having mental health problems (Biringer et al., 2015; Klemetti et al., 2010; Verhaak et al., 2007). This hypothesis could be supported by our observation that MAR children were at higher risk of having internalizing disorders (depression and anxiety) in particular. Second, parenting behaviors such as overprotectiveness, higher level of parental stress, or higher parental expectations in MAR families could negatively influence the parent–child relationship and, as a consequence, the children’s psychosocial development (Bernstein, 1990; Colpin & Soenen, 2002; Golombok et al., 1995; Wagenaar et al., 2008, 2009). Along the same lines, it has been suggested that the disclosure of their mode of conception may induce stress and potential difficulties in psychological adjustment for MAR adolescents (Ilioi & Golombok, 2015; Tallandini et al., 2016). However, the limited existing evidence does not fully support these hypotheses, as it shows that MAR adolescents are well adjusted and have positive parent–child relationships (Goisis & Palma, 2021; Ilioi & Golombok, 2015). Third, an additional potential explanation for indications that MAR adolescents have worse mental health could be related to different patterns of treatment seeking. MAR parents are, on average, more advantaged, which is associated with a higher probability of health care use, even in a country like Finland with extensive, highly state-subsidized public health care (Blomgren & Virta, 2020; Evans-Lacko et al., 2018; Hodgkinson et al., 2017). While the within-family models should account for an overall tendency for treatment seeking in families, parents might still differ in their approaches to their children depending on whether they were conceived through MAR or naturally.

Taken together, our findings highlight two main aspects. First, while the population-level results show a positive picture for MAR-conceived children, a potentially negative effect of MAR on mental health outcomes remains after we take family characteristics into account. Although relatively small, this effect merits further investigation to uncover the underlying mechanisms. Second, the selection process into MAR is highly integral to the differences: The positively selected characteristics of MAR parents (Goisis et. al 2020) result in MAR-conceived children outperforming naturally conceived children on social outcomes, and compensating for their otherwise increased risk of mental health disorders. The comprehensive perspective on MAR children that this study provides for the first time highlights a potential trade-off and variation in the link between MAR and adolescent outcomes across different dimensions of well-being.

### Strengths and Weaknesses

Our study was based on administrative total population data on a total of five birth cohorts who could be followed up to age 18. Thus, our analyses were not marred by non-representativeness, self-report bias, or a lack of statistical power, even when studying relatively rare outcomes. For the rarest of our study outcomes (externalizing, developmental disorders, and high-risk health behaviours, in particular), we would like to nevertheless emphasize the need for a cautious interpretation of the associations, or a lack of them. The exclusion of individuals with missing data on covariates (4%) is unlikely to seriously bias our results, but may have led to somewhat more conservative estimates on the differentials, as these adolescents are likely to represent a socially more disadvantaged group.

Children conceived through MAR were identified through register data on medication purchases. Moreover, unlike many previous studies that focused on IVF, we also had data on less invasive treatments, such as ovulation induction, that have been previously shown to be associated with children’s later outcomes (Bay et al., 2013). While our main focus was on the outcomes of adolescents conceived through MAR, regardless of the treatment type, we replicated our analyses on IVF births to better enable a comparison of our results with those of the previous literature. The medication data did not allow us to classify all MAR births according to the type of treatment, and a comparison of our findings with those of Hemminki et al. (2003) indicated that we underestimated the proportion of IVF births by 10%. While this is a limitation of our study, the results of the analyses on IVF were close to those of our main analyses, and the relatively small proportion of missing cases of IVF is unlikely to cause serious bias in the IVF-specific results.

Using administrative register data, we were able to assess a variety of late adolescent outcomes, some of which are new to the existing literature on MAR-conceived children (NEET status, early home-leaving, antidepressant use, and high-risk health behaviors). The measures of mental disorders and high-risk health behaviors are based on health conditions that have been treated and are thus likely to reflect the more severe end of mental health problems. While the overall high quality and representativeness of the administrative health care data have been previously reviewed (Sund, 2012), as is the case with any data based on health care records, the potential under-coverage of mental health problems due to not seeking treatment remains an issue.

To our knowledge, this is the first study on longer-term outcomes among MAR- and naturally conceived children to take advantage of sibling comparisons to adjust for unobserved confounding by family background. The within-family analyses complemented our results pertaining to the overall population and confirmed the important role of selection by family background in explaining differences by mode of conception. Nonetheless, however, valuable sibling fixed-effects models are in eradicating any effects of shared environment and genetic endowment, they have their limitations as well. In our data, the within-family sample of MAR-conceived children with naturally conceived siblings represented a minority of the full sample; i.e., less than a fifth of all MAR adolescents. Although there were no major differences between the MAR adolescents or their parents in the total sample and the within-family sample, the parents who were able to conceive naturally likely suffered from less severe subfertility. We were also not able to control for potentially important factors not shared by siblings, such as personality, cognitive skills, or differences in parent–child relationships. Finally, the actual mechanisms through which mode of conception may induce differences between siblings remain essentially a black box. Future studies with self-reported data on parental behaviors, expectations and attitudes—ideally on multiple children within the same family—might shed light on the potential mechanisms at play. We also encourage future longitudinal studies to delve deeper into the emergence and development of mental disorders among MAR-conceived children using a life course approach.

There are notable between-country differences in access to infertility treatment and the prevalence of MAR (De Geyter et al., 2020), Thus, while our findings are nationally representative, the extent to which they are generalizable remains an open question. Despite the differences in prevalence, selection into MAR nevertheless appears to be similar across contexts: i.e., women and couples who suffer from subfertility and seek MAR treatment tend to have more or less advantaged socioeconomic backgrounds, even in countries where fertility treatments are highly state-subsidized (Chambers et al., 2014; Chandra et al., 2014; Goisis et al., 2020). Therefore, we could expect the direction of the associations between MAR and the outcomes analyzed in this study to be similar across contexts, while the strength of the associations could vary. However, among population subgroups, processes of selection into fertility treatment may differ more strongly. For example, there have been no major changes in the overall availability of treatments in Finland, but in the late 1990s when the cohorts included in this study were born, public-sector fertility treatments were not yet offered to single women or same-sex couples. A replication of this study using data from a variety of contexts is warranted.

## Conclusions

The future prevalence of MAR is likely to increase in wealthy countries, of which many have been faced with significant fertility declines, postponement of childbearing, and increasing diversity in family forms within the last decades (Esping-Andersen & Billari, 2015). Our uniquely rich data allowed us to probe several dimensions of well-being among contemporary cohorts of MAR-conceived adolescents as they approached the transition to adulthood. Our population-level findings on their longer-term social and mental health outcomes are mostly reassuring. However, our observation that MAR adolescents had a higher probability of experiencing mental health problems once we accounted for family sociodemographic characteristics is a potential cause for concern and merits further attention. As the numbers of MAR children reaching late adolescence and early adulthood are increasing, it is important to continue to monitor these individuals to assess their specific conditions, and the severity and persistence of these conditions across the life course. In addition, future research should seek to identify the mechanisms through which mental health problems among MAR-conceived children emerge during childhood and adolescence.

## Data Availability

The study uses data that are collected by register authorities (Statistics Finland, the Finnish Institute for Health and Welfare, the Social Insurance Institution) and made available to researchers under license for scientific research specified in a research plan. All data used or produced by combining original data are confidential, and the researchers cannot share them with third parties.

## Appendix

See Tables

**Table 4:** ICD-10 codes and prevalence (%) of different types of health conditions at ages 16–18 within the broader groups of mental disorders and high-risk health behaviour

Health condition group	ICD-10 codes	%
*Mental disorders (any)*	F20–F69, F80–F99^a^	9.27
*Internalizing*		5.92
Depression	F32–F33^b^	3.63
Anxiety	F40–F42	3.27
*Externalizing*		1.27
Attention deficit and hyperactivity disorder/hyperkinetic disorder	F90	0.77
Conduct disorder	F91–F92	0.56
*Developmental*	F80–F89	1.03
*Other mental disorders*		3.94
Schizophrenia, schizotypal, and delusional disorders	F20–F29	0.39
Mood disorders	F30–F39 ^c^	0.62
Neurotic, stress-related, and somatoform disorders	F43–F48	1.05
Behavioral syndromes associated with physiological disturbances and physical factors	F50–F59	0.89
Disorders of adult personality and behavior	F60–F69	0.24
Disorders with onset usually occurring in childhood and adolescence; unspecified	F93–F99	1.28
*High-risk health behaviour*		1.85
Mental and behavioural disorders due to psychoactive substance use	F10–F16, F18–F19	1.07
Poisoning by drugs, medications, and alcohol	T36–T51, X44–45	0.27
Intentional self-harm	X60–X84, Y870	0.38
Assaults	X85-99, Y20-29, Y871	0.40

^a^Excluding F00–F09 (organic mental disorders), F10–F19 (psychoactive substance use, included in high-risk health behavior), and F70–F79 (mental retardation)

^b^Excluding codes F32.3 and F33.3 (depression with psychotic features)

^c^Excluding codes F32 and F33 included in depression

The prevalence in the sub-categories does not sum up to the higher level, as individuals may have several diagnoses

4,

**Table 5:** Differences in the probability (percentage points) of social and mental-health outcomes at ages 16–18 among children conceived by in vitro fertilization (IVF) versus those conceived naturally. Between-family and within-family analyses of children born in Finland 1995−2000

IVF	Between-family sample			Within-family sample		
	Model 1	Model 2	N	Model 3	Model 4	N
Grade point average (GPA) ^a^	31.60*** (1.567)	9.192*** (1.435)	213,317	6.322 (5.818)	− 9.006 (10.51)	555
Academic track (ref. vocational)	13.73*** (0.675)	2.871*** (0.624)	261,720	7.550** (2.390)	0.588 (3.853)	952
School dropout	− 0.745** (0.231)	− 0.084 (0.232)	272,485	− 0.580 (0.788)	− 0.896 (1.260)	986
NEET status at age 18	− 1.262*** (0.253)	− 0.086 (0.253)	272,485	− 0.225 (0.726)	1.427 (1.157)	986
Early home-leaving	− 7.078*** (0.505)	− 1.798*** (0.492)	272,485	− 1.796 (1.718)	1.050 (2.753)	986
Antidepressant use	− 0.719* (0.356)	− 0.0360 (0.358)	272,485	1.250 (1.630)	− 1.033 (2.615)	986
Mental disorders (any)	− 0.728 (0.391)	0.338 (0.392)	272,485	2.163 (1.580)	− 0.733 (2.497)	986
Internalizing	− 0.330 (0.317)	0.278 (0.319)	272,485	1.255 (1.424)	− 1.933 (2.259)	986
Externalizing	− 0.449** (0.152)	0.029 (0.153)	272,485	0.908 (0.643)	0.467 (1.037)	986
Developmental	0.180 (0.136)	0.295* (0.138)	272,485	− 0.088 (0.580)	− 0.847 (0.925)	986
Other mental disorders	− 0.189 (0.263)	0.148 (0.265)	272,485	1.343 (1.153)	− 2.122 (1.812)	986
High-risk health behaviour	− 0.432* (0.183)	0.071 (0.184)	272,485	− 0.869 (0.656)	− 0.473 (1.047)	986
Adjustments						
Child's sex	x	x		x	x	
Birth year	x	x				
Birth order		x			x	
Maternal age at birth		x				
Maternal smoking		x			x	
Parental education at birth		x				
Family structure at birth		x				
Income decile at birth		x			x	
Hospital district at birth		x				

Standard Errors in parentheses. Sample sizes vary across outcomes, but are held constant for each outcome across estimations for each type of analysis. Sibling fixed effects used for the within-family models. Significance levels: ****p* < 0.001, ***p* < 0.01, **p* < 0.05

^a^The coefficient (multiplied by 100) for GPA represents a linear estimation, not a probability

5,

**Table 6:** Differences in the probability (percentage points) of social and mental-health outcomes at ages 16–18 among children conceived by any medically assisted reproduction (MAR) and in vitro fertilization (IVF) versus those conceived naturally. Between-family and within-family analyses of children born in Finland 1995−2000, adjusted for sociodemographic factors and multiple births, LBW, and gestational age

	MAR children compared to naturally conceived children		IVF children compared to naturally conceived children	
	Between-family sample	Within-family sample	Between-family sample	Within-family sample
	Model 2a	Model 4a	Model 2a	Model 4a
Grade point average (GPA) ^a^	3.464*** (0.957)	1.005 (3.465)	7.339*** (1.495)	− 0.079 (0.109)
Academic track (ref. vocational)	1.080** (0.418)	0.427 (1.351)	2.451*** (0.651)	0.002 (0.041)
School dropout	− 0.036 (0.155)	− 0.077 (0.471)	− 0.182 (0.242)	− 0.013 (0.013)
NEET status at age 18	− 0.054 (0.168)	− 0.457 (0.506)	0.049 (0.263)	0.013 (0.012)
Early home-leaving	− 1.667*** (0.328)	0.452 (0.956)	− 1.811*** (0.513)	0.013 (0.029)
Antidepressant use	0.515* (0.240)	0.573 (0.866)	0.033 (0.373)	0.014 (0.028)
Mental disorders (any)	0.745** (0.263)	1.184 (0.914)	0.438 (0.409)	− 0.003 (0.027)
Internalizing	0.627** (0.214)	1.154 (0.754)	0.449 (0.333)	− 0.006 (0.024)
Externalizing	0.099 (0.102)	− 0.203 (0.342)	0.032 (0.159)	0.001 (0.011)
Developmental	0.263** (0.0925)	− 0.055 (0.350)	0.210 (0.144)	− 0.010 (0.001)
Other mental disorders	0.158 (0.177)	0.326 (0.663)	0.060 (0.276)	− 0.022 (0.019)
High-risk health behaviour	0.156 (0.123)	0.238 (0.444)	0.079 (0.192)	− 0.005 (0.011)
Adjustments:				
Child's sex	x	x	x	x
Birth year	x		x	
Birth order	x	x	x	x
Maternal age at birth	x		x	
Maternal smoking	x	x	x	x
Parental education at birth	x		x	
Family structure at birth	x		x	
Income decile at birth	x	x	x	x
Hospital district at birth	x		x	
Multiple birth	x	x	x	x
Low birth weight (LBW)	x	x	x	x
Gestational age (days)	x	x	x	x

Standard Errors in parentheses. Sample sizes vary across outcomes but are held constant for each outcome across estimations for each type of analysis. Sample sizes of each outcome are the same as reported in Table 3 and Table 5. Sibling fixed effects used for the within-family models. Significance levels: ****p* < 0.001, ***p* < 0.01, **p* < 0.05

^a^The coefficient (multiplied by 100) for GPA represents a linear estimation, not a probability

6,

**Table 7:** Differences in the probability (percentage points) of social and mental-health outcomes at ages 16–18 among singleton children conceived by medically assisted reproduction (MAR) versus those conceived naturally. Between-family and within-family analyses of singleton children born in Finland 1995−2000, adjusted for sociodemographic factors

MAR	Between− family sample			Within− family sample		
	Model 1	Model 2	*N*	Model 3	Model 4	*N*
Grade point average (GPA) ^a^	23.40*** (1.141)	3.217*** (1.046)	212,611	3.661 (2.965)	1.224 (3.562)	2446
Academic track (ref. vocational)	10.49*** (0.493)	0.850* (0.456)	261,030	2.580* (1.157)	0.399 (1.376)	4375
School dropout	− 0.630*** (0.168)	0.0387 (0.169)	271,710	− 0.096 (0.404)	0.285 (0.477)	4603
NEET status at age 18	− 1.088*** (0.184)	0.001 (0.184)	271,710	− 0.678 (0.450)	− 0.315 (0.532)	4603
Early home-leaving	− 6.232*** (0.367)	− 1.622*** (0.359)	271,710	− 0.804 (0.831)	0.590 (0.984)	4603
Antidepressant use	− 0.149 (0.260)	0.552** (0.262)	271,710	0.216 (0.755)	0.582 (0.893)	4603
Mental disorders (any)	− 0.278 (0.285)	0.781*** (0.287)	271,710	2.187** (0.790)	1.357 (0.936)	4603
Internalizing	0.013 (0.232)	0.653*** (0.234)	271,710	1.725** (0.653)	1.185 (0.774)	4603
Externalizing	− 0.273* (0.111)	0.135 (0.112)	271,710	0.234 (0.273)	− 0.027 (0.323)	4603
Developmental	0.203* (0.010)	0.321*** (0.101)	271,710	0.289 (0.306)	− 0.003 (0.362)	4603
Other mental disorders	− 0.246 (0.192	0.106 (0.193)	271,710	0.809 (0.576)	0.237) (0.680)	4603
High-risk health behaviour	− 0.242 (0.133)	0.207 (0.134)	271,710	0.292 (0.396)	0.180) (0.469)	4603
Adjustments:						
Child's sex	x	x		x	x	
Birth year	x	x				
Birth order		x			x	
Maternal age at birth	x					
Maternal smoking	x			x		
Parental education at birth	x					
Family structure at birth	x					
Income decile at birth	x			x		
Hospital district at birth	x					

Standard Errors in parentheses. Sample sizes vary across outcomes but are held constant for each outcome across estimations for each type of analysis. Sibling fixed effects used for the within-family models. Significance levels: ****p* < 0.001, ***p* < 0.01, **p* < 0.05

^a^The coefficient (multiplied by 100) for GPA represents a linear estimation, not a probability

7,

**Table 8:** Differences in the probability (percentage points) of social and mental-health outcomes at ages 16–18 among boys conceived by medically assisted reproduction (MAR) versus those conceived naturally. Between-family and within-family analyses of boys born in Finland 1995−2000

MAR	Between− family sample		Within− family sample	
	Model 1	Model 2	Model 3	Model 4
Grade point average (GPA) ^a^	24.15*** (1.414)	5.375*** (1.290)	6.693 (4.990)	− 1.138 (5.386)
Academic track (ref. vocational)	10.53*** (0.609)	1.711*** (0.561)	3.767** (1.922)	1.537 (2.083)
School dropout	− 0.931*** (0.208)	− 0.316 (0.208)	− 1.076 (0.673)	− 1.035 (0.732)
NEET status at age 18	− 1.496*** (0.227)	− 0.482** (0.226)	− 0.813 (0.724)	− 1.040 (0.787)
Early home-leaving	− 4.874*** (0.454)	− 0.445 (0.441)	0.585 (1.364)	− 0.109 (1.483)
Antidepressant use	− 0.381 (0.321)	0.281 (0.322)	0.439 (1.238)	− 0.184 (1.345)
Mental disorders (any)	− 0.446 (0.353)	0.563 (0.353)	1.503 (1.307)	0.191 (1.420)
Internalizing	− 0.165 (0.287)	0.437 (0.288)	0.936 (1.076)	− 0.157 (1.170)
Externalizing	− 0.441*** (0.137)	− 0.042 (0.137)	− 0.402 (0.489)	− 0.697 (0.532)
Developmental	0.239* (0.124)	0.345*** (0.124)	1.079** (0.500)	0.844 (0.543)
Other mental disorders	− 0.457* (0.237)	− 0.123 (0.238)	− 0.268 (0.949)	− 0.535 (1.030)
High-risk health behaviour	− 0.350** (0.165)	0.087 (0.165)	0.408 (0.633)	0.053 (0.690)
Adjustments:				
Child's sex	x	x	x	x
Birth year	x	x		
Birth order		x		x
Maternal age at birth		x		
Maternal smoking		x		x
Parental education at birth		x		
Family structure at birth		x		
Income decile at birth		x		x
Hospital district at birth		x		

Standard Errors in parentheses. Sample sizes vary across outcomes but are held constant for each outcome across estimations for each type of analysis. Sample sizes of each outcome are the same as reported in Table 3. Sibling fixed effects used for the within-family models. Significance levels: ****p* < 0.001, ***p* < 0.01, **p* < 0.05

^a^The coefficient (multiplied by 100) for GPA represents a linear estimation, not a probability

8,

**Table 9:** Differences in the probability (percentage points) of social and mental-health outcomes at ages 16–18 among girls conceived by medically assisted reproduction (MAR) versus those conceived naturally. Between-family and within-family analyses of girls born in Finland 1995−2000

MAR	Between− family sample		Within− family sample	
	Model 1	Model 2	Model 3	Model 4
Grade point average (GPA) ^a^	23.93*** (1.434)	4.132*** (1.309)	− 2.387 (5.009)	− 8.403 (5.399)
Academic track (ref. vocational)	10.46*** (0.624)	1.010* (0.575)	− 0.337 (1.978)	− 2.320 (2.137)
School dropout	− 0.276 (0.213)	0.384* (0.213)	1.317* (0.689)	1.425* (0.746)
NEET status at age 18	− 0.857*** (0.232)	0.223 (0.231)	0.339 (0.741)	0.149 (0.802)
Early home-leaving	− 7.586*** (0.464)	− 3.001*** (0.451)	− 0.469 (1.396)	− 0.972 (1.511)
Antidepressant use	− 0.0670 (0.328)	0.607* (0.329)	− 0.614 (1.268)	− 1.393 (1.371)
Mental disorders (any)	− 0.187 (0.361)	0.829** (0.361)	1.457 (1.338)	− 0.004 (1.447)
Internalizing	− 0.0112 (0.293)	0.588** (0.294)	1.348 (1.102)	0.114 (1.192)
Externalizing	− 0.147 (0.140)	0.260* (0.140)	0.631 (0.501)	0.223 (0.542)
Developmental	0.177 (0.126)	0.285** (0.127)	− 0.976* (0.512)	− 1.230** (0.554)
Other mental disorders	0.232 (0.242)	0.579** (0.244)	1.219 (0.971)	0.843 (1.049)
High-risk health behaviour	− 0.257 (0.168)	0.196 (0.169)	0.0108 (0.648)	− 0.323 (0.703)
Adjustments:				
Child's sex	x	x	x	x
Birth year	x	x		
Birth order		x		x
Maternal age at birth		x		
Maternal smoking		x		x
Parental education at birth		x		
Family structure at birth		x		
Income decile at birth		x		x
Hospital district at birth		x		

Standard Errors in parentheses. Sample sizes vary across outcomes but are held constant for each outcome across estimations for each type of analysis. Sample sizes of each outcome are the same as reported in Table 3. Sibling fixed effects used for the within-family models. Significance levels: ****p* < 0.001, ***p* < 0.01, **p* < 0.05

^a^The coefficient (multiplied by 100) for GPA represents a linear estimation, not a probability

9.
