# Correction: The Well-Being of Adolescents Conceived Through Medically Assisted Reproduction: A Population-Level and Within-Family Analysis

**DOI:** 10.1007/s10680-023-09663-6

**Published:** 2023-04-11

**Authors:** Hanna Remes, Maria Palma Carvajal, Riina Peltonen, Pekka Martikainen, Alice Goisis

**Affiliations:** 1 Population Research Unit, Faculty of Social Sciences, University of Helsinki, P.O. Box 18, 00014 Helsinki, Finland; 2 University College London, Social Research Institute, London, UK; 3 Department of Public Health Sciences, Stockholm University, Stockholm, Sweden; 4 Laboratory of Population Health, Max Planck Institute for Demographic Research, Rostock, Germany

**Correction to: European Journal of Population (2022) 38:915–949** 10.1007/s10680-022-09623-6

In this article, all the standard errors (i.e., all figures in parentheses) in Tables 3, 5 and 7 contain minus signs that should not be there. The correct tables are shown in the following.

**Table 3:** Differences in the probability (percentage points) of social and mental-health outcomes at ages 16–18 among children conceived by medically assisted reproduction (MAR) versus those conceived naturally. Between-family and within-family analyses of children born in Finland 1995–2000

MAR	Between-family sample			Within-family sample		
	Model 1	Model 2	*n*	Model 3	Model 4	*n*
Grade point average (GPA)^a^	24.04***	4.763***	219,624	3.732	1.295	2621
	(1.008)	(0.925)		(2.809)	(3.397)	
Academic track (ref. vocational)	10.50***	1.369***	269,662	3.081**	0.786	4682
	(0.436)	(0.405)		(1.107)	(1.324)	
School dropout	− 0.611***	0.026	280,682	− 0.114	0.040	4923
	(0.149)	(0.150)		(0.388)	(0.462)	
NEET status at age 18	− 1.184***	− 0.137	280,682	− 0.603	− 0.357	4923
	(0.163)	(0.163)		(0.417)	(0.496)	
Early home-leaving	− 6.201***	− 1.695***	280,682	− 1.040	0.342	4923
	(0.325)	(0.318)		(0.786)	(0.935)	
Antidepressant use	− 0.227	0.440	280,682	− 0.146	0.193	4923
	(0.230)	(0.232)		(0.713)	(0.848)	
Mental disorders (any)	− 0.319	0.693**	280,682	2.124**	1.177	4923
	(0.252)	(0.254)		(0.752)	(0.895)	
Internalizing	− 0.090	0.511*	280,682	1.556*	0.928	4923
	(0.205)	(0.207)		(0.620)	(0.738)	
Externalizing	− 0.297**	0.106	280,682	0.257	− 0.072	4923
	(0.098)	(0.099)		(0.282)	(0.335)	
Developmental	0.209*	0.315***	280,682	0.329	0.037	4923
	(0.088)	(0.090)		(0.288)	(0.343)	
Other	− 0.120	0.220	280,682	1.002	0.395	4923
	(0.170)	(0.172)		(0.546)	(0.649)	
High-risk health behavior	− 0.304**	0.140	280,682	0.234	0.185	4923
	(0.118)	(0.119)		(0.365)	(0.435)	
Adjustments:						
Child's sex	x	x		x	x	
Birth year	x	x				
Birth order		x			x	
Maternal age at birth		x				
Maternal smoking		x			x	
Parental education at birth		x				
Family structure at birth		x				
Income decile at birth		x			x	
Hospital district at birth		x				

Standard errors in parentheses. Sibling fixed effects used for the within-family models. Significance levels: ***p < 0.001, **p < 0.01, *p < 0.05

^a^The coefficient (multiplied by 100) for GPA represents a linear estimation, not a probability

**Table 5:** Differences in the probability (percentage points) of social and mental-health outcomes at ages 16–18 among children conceived by in vitro fertilization (IVF) versus those conceived naturally. Between-family and within-family analyses of children born in Finland 1995–2000

IVF	Between-family sample			Within-family sample		
	Model 1	Model 2	N	Model 3	Model 4	N
Grade point average (GPA)^a^	31.60***	9.192***	213,317	6.322	− 9.006	555
	(1.567)	(1.435)		(5.818)	(10.51)	
Academic track (ref. vocational)	13.73***	2.871***	261,720	7.550**	0.588	952
	(0.675)	(0.624)		(2.390)	(3.853)	
School dropout	− 0.745**	− 0.084	272,485	− 0.580	− 0.896	986
	(0.231)	(0.232)		(0.788)	(1.260)	
NEET status at age 18	− 1.262***	− 0.086	272,485	− 0.225	1.427	986
	(0.253)	(0.253)		(0.726)	(1.157)	
Early home-leaving	− 7.078***	− 1.798***	272,485	− 1.796	1.050	986
	(0.505)	(0.492)		(1.718)	(2.753)	
Antidepressant use	− 0.719*	− 0.0360	272,485	1.250	− 1.033	986
	(0.356)	(0.358)		(1.630)	(2.615)	
Mental disorders (any)	− 0.728	0.338	272,485	2.163	− 0.733	986
	(0.391)	(0.392)		(1.580)	(2.497)	
Internalizing	− 0.330	0.278	272,485	1.255	− 1.933	986
	(0.317)	(0.319)		(1.424)	(2.259)	
Externalizing	− 0.449**	0.029	272,485	0.908	0.467	986
	(0.152)	(0.153)		(0.643)	(1.037)	
Developmental	0.180	0.295*	272,485	− 0.088	− 0.847	986
	(0.136)	(0.138)		(0.580)	(0.925)	
Other	− 0.189	0.148	272,485	1.343	− 2.122	986
	(0.263)	(0.265)		(1.153)	(1.812)	
High-risk health behavior	− 0.432*	0.071	272,485	− 0.869	− 0.473	986
	(0.183)	(0.184)		(0.656)	(1.047)	
Adjustments:						
Child's sex	x	x		x	x	
Birth year	x	x				
Birth order		x			x	
Maternal age at birth		x				
Maternal smoking		x			x	
Parental education at birth		x				
Family structure at birth		x				
Income decile at birth		x			x	
Hospital district at birth		x				

Standard errors in parentheses. Sample sizes vary across outcomes but are held constant for each outcome across estimations for each type of analysis. Sibling fixed effects used for the within-family models. Significance levels: ***p < 0.001, **p < 0.01, *p < 0.05

^a^The coefficient (multiplied by 100) for GPA represents a linear estimation, not a probability

**Table 7:** Differences in the probability (percentage points) of social and mental-health outcomes at ages 16–18 among singleton children conceived by medically assisted reproduction (MAR) versus those conceived naturally. Between-family and within-family analyses of singleton children born in Finland 1995–2000, adjusted for sociodemographic factors

MAR	Between-family sample			Within-family sample		
	Model 1	Model 2	*N*	Model 3	Model 4	*N*
Grade point average (GPA)^a^	23.40***	3.217***	212,611	3.661	1.224	2446
	(1.141)	(1.046)		(2.965)	(3.562)	
Academic track (ref. vocational)	10.49***	0.850*	261,030	2.580*	0.399	4375
	(0.493)	(0.456)		(1.157)	(1.376)	
School dropout	− 0.630***	0.0387	271,710	− 0.096	0.285	4603
	(0.168)	(0.169)		(0.404)	(0.477)	
NEET status at age 18	− 1.088***	0.001	271,710	− 0.678	− 0.315	4603
	(0.184)	(0.184)		(0.450)	(0.532)	
Early home-leaving	− 6.232***	− 1.622***	271,710	− 0.804	0.590	4603
	(0.367)	(0.359)		(0.831)	(0.984)	
Antidepressant use	− 0.149	0.552**	271,710	0.216	0.582	4603
	(0.260)	(0.262)		(0.755)	(0.893)	
Mental disorders (any)	− 0.278	0.781***	271,710	2.187**	1.357	4603
	(0.285)	(0.287)		(0.790)	(0.936)	
Internalizing	0.013	0.653***	271,710	1.725**	1.185	4603
	(0.232)	(0.234)		(0.653)	(0.774)	
Externalizing	− 0.273*	0.135	271,710	0.234	− 0.027	4603
	(0.111)	(0.112)		(0.273)	(0.323)	
Developmental	0.203*	0.321***	271,710	0.289	− 0.003	4603
	(0.010)	(0.101)		(0.306)	(0.362)	
Other	− 0.246	0.106	271,710	0.809	0.237)	4603
	(0.192	(0.193)		(0.576)	(0.680)	
High-risk health behavior	− 0.242	0.207	271,710	0.292	0.180)	4603
	(0.133)	(0.134)		(0.396)	(0.469)	
Adjustments:						
Child's sex	x	x		x	x	
Birth year	x	x				
Birth order		x			x	
Maternal age at birth		x				
Maternal smoking		x			x	
Parental education at birth		x				
Family structure at birth		x				
Income decile at birth		x			x	
Hospital district at birth		x				

Standard errors in parentheses. Sample sizes vary across outcomes but are held constant for each outcome across estimations for each type of analysis. Sibling fixed effects used for the within-family models. Significance levels: ***p < 0.001, **p < 0.01, *p < 0.05

^a^The coefficient (multiplied by 100) for GPA represents a linear estimation, not a probability

The original article has been corrected.

